# Reintubation Summation Calculation: A Predictive Score for Extubation Failure in Critically Ill Patients

**DOI:** 10.3389/fmed.2021.789440

**Published:** 2022-02-17

**Authors:** Vikas Bansal, Nathan J. Smischney, Rahul Kashyap, Zhuo Li, Alberto Marquez, Daniel A. Diedrich, Jason L. Siegel, Ayan Sen, Amanda D. Tomlinson, Carla P. Venegas-Borsellino, William David Freeman

**Affiliations:** ^1^Division of Pulmonary and Critical Care Medicine, Department of Medicine, Mayo Clinic, Rochester, MN, United States; ^2^Critical Care Independent Multidisciplinary Program, Mayo Clinic, Rochester, MN, United States; ^3^Department of Anesthesiology and Perioperative Medicine, Mayo Clinic, Rochester, MN, United States; ^4^Biostatistics Unit, Mayo Clinic, Jacksonville, FL, United States; ^5^Department of Critical Care Medicine, Mayo Clinic, Jacksonville, FL, United States; ^6^Department of Neurologic Surgery, Mayo Clinic, Jacksonville, FL, United States; ^7^Department of Neurology, Mayo Clinic, Jacksonville, FL, United States; ^8^Department of Critical Care Medicine, Mayo Clinic Hospital, Phoenix, AZ, United States; ^9^Department of Neurologic Surgery, Mayo Clinic Hospital, Phoenix, AZ, United States

**Keywords:** critical care medicine, extubation failure, intensive care unit, mechanical ventilation, reintubation, predictive modeling, prediction scale

## Abstract

**Objective:**

To derive and validate a multivariate risk score for the prediction of respiratory failure after extubation.

**Patients and methods:**

We performed a retrospective cohort study of adult patients admitted to the intensive care unit from January 1, 2006, to December 31, 2015, who received mechanical ventilation for ≥48 h. Extubation failure was defined as the need for reintubation within 72 h after extubation. Multivariate logistic regression model coefficient estimates generated the **R**e-**I**ntubation **S**ummation **C**alculation (RISC) score.

**Results:**

The 6,161 included patients were randomly divided into 2 sets: derivation (*n* = 3,080) and validation (*n* = 3,081). Predictors of extubation failure in the derivation set included body mass index <18.5 kg/m^2^ [odds ratio (OR), 1.91; 95% CI, 1.12–3.26; *P* = 0.02], threshold of Glasgow Coma Scale of at least 10 (OR, 1.68; 95% CI, 1.31–2.16; *P* < 0.001), mean airway pressure at 1 min of spontaneous breathing trial <10 cmH_2_O (OR, 2.11; 95% CI, 1.68–2.66; *P* < 0.001), fluid balance ≥1,500 mL 24 h preceding extubation (OR, 2.36; 95% CI, 1.87–2.96; *P* < 0.001), and total mechanical ventilation days ≥5 (OR, 3.94; 95% CI 3.04–5.11; *P* < 0.001). The C-index for the derivation and validation sets were 0.72 (95% CI, 0.70–0.75) and 0.72 (95% CI, 0.69–0.75). Multivariate logistic regression demonstrated that an increase of 1 in RISC score increased odds of extubation failure 1.6-fold (OR, 1.58; 95% CI, 1.47–1.69; *P* < 0.001).

**Conclusion:**

RISC predicts extubation failure in mechanically ventilated patients in the intensive care unit using several clinically relevant variables available in the electronic medical record but requires a larger validation cohort before widespread clinical implementation.

## Introduction

Before extubating a mechanically ventilated patient, intensivists must evaluate the patient's risk of extubation failure (EF). This decision is usually based on the results of a rapid shallow breathing index (RSBI), which is most often assessed during the readiness evaluation to identify patients who may proceed with the spontaneous breathing trial (SBT), with either a T-piece or low-level pressure support (1–3). The RSBI is the ratio of respiratory frequency to tidal volume and is a commonly used weaning predictor (1–3). This method is not 100% predictive of extubation success (ES) by 72 h. The decision-making behind extubation is critically important, as failed extubation occurs in 10–20% of intensive care unit (ICU) patients (4, 5) and both delayed extubation as well as early extubation are associated with worse outcomes. Extubation delay is associated with ventilator-associated pneumonia (6, 7), increased length of stay, increased risk for downstream tracheostomy (8, 9) and increased mortality in brain-injured patients (8). Extubation failure after planned extubation is associated with adverse outcomes including increased hospital mortality, prolonged hospital stay, higher costs, and greater need for tracheotomy and transfer to post-acute care (10–13).

While there are numerous ventilator weaning predictors and types of SBTs (14, 15), there is a paucity of data on risk factors that predict EF prior to removal of the endotracheal tube. Predicting factors for ES and EF include amount of endotracheal secretions (8, 16, 17), cough strength (16, 18, 19), and mental status prior to extubation after a successful SBT (16, 20). Patients with moderate or abundant secretions have been 3–8 times more likely to fail extubation than those with few to no secretions (8, 19). Coplin et al. (16) stated that the Glasgow coma scale (GCS) score alone did not predict extubation outcome in brain-injured patients and should not be used to exclude extubation; however, other investigators reported that impaired mental status did predict EF (21, 22). Moreover, patients who fail extubation often retain carbon dioxide because of an imbalance among respiratory muscle strength and imposed load (1, 23–25). Patients extubated while developing hypercapnia (Paco_2_ >45 mmHg) during a successful SBT may also have an increased risk of mortality due to respiratory failure compared to those who do not develop hypercapnia during SBT (2).

Therefore, we hypothesized that recurrent respiratory failure requiring reintubation after initial extubation could be estimated using a composite score of known risk factors available in the electronic medical record (EMR). Our main objective was to derive a simple clinical prediction tool using a multivariate model and validate the **R**e-**I**ntubation **S**cale **C**alculation (RISC) score to predict respiratory failure requiring reintubation.

## Methods

### Study Population

**Figure 1:**
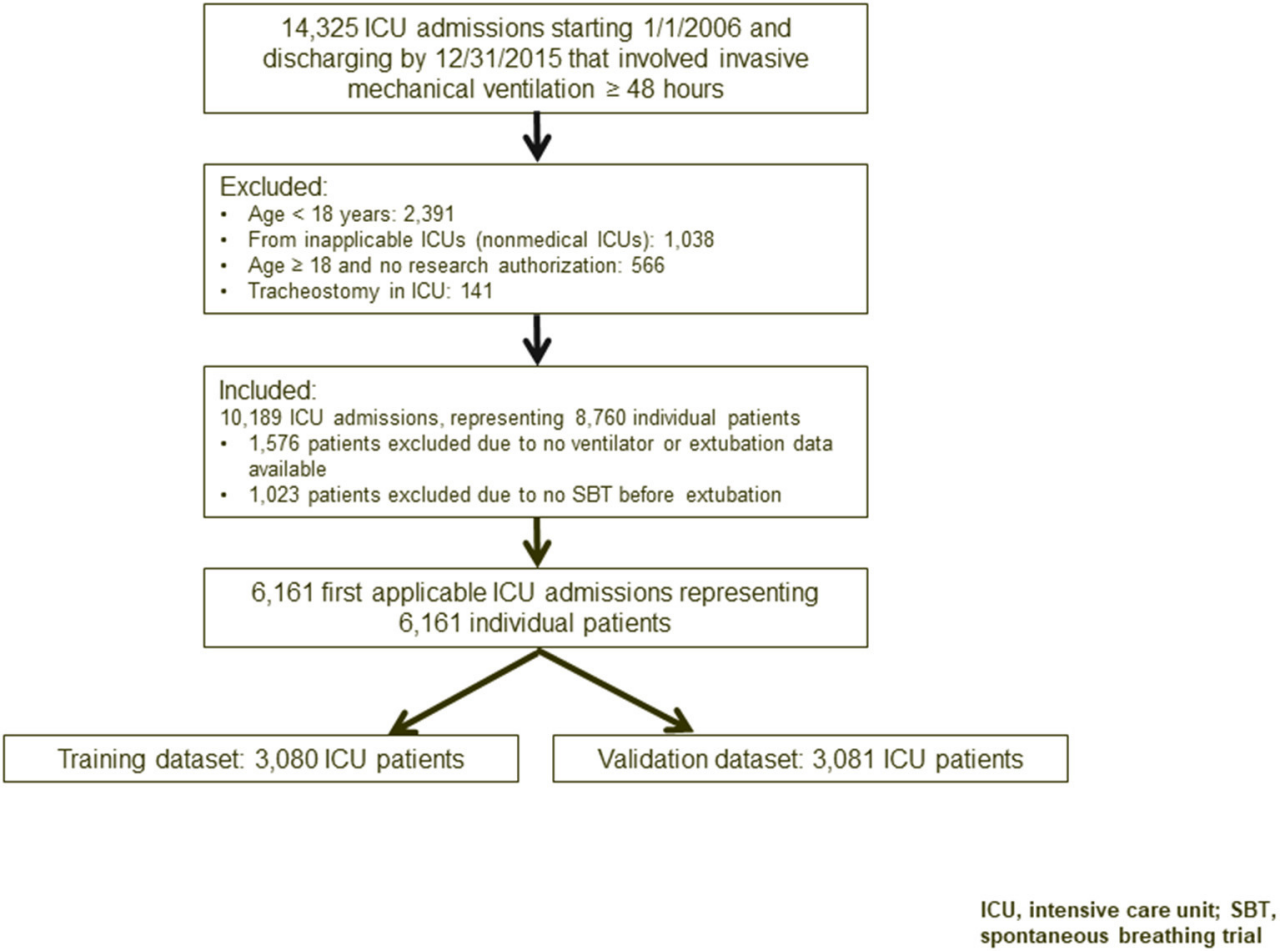
Flowchart of study participants. ICU, intensive care unit; SBT, spontaneous breathing trial.

We performed a retrospective cohort study to develop and validate the RISC score. Our study population included critically ill adults who were on mechanical ventilation for ≥48 h during their stay in a medical, surgical, or mixed ICU between January 1, 2006, and December 31, 2015 (Figure 1). The study was approved by the Mayo Clinic Institutional Review Board for the use of existing medical records of patients with prior research authorization.

### Inclusion Criteria

We gathered data for patients who met the following criteria: age ≥18 years; intubation and mechanical ventilation for ≥48 h; adequate oxygenation, suggested by Pao_2_ >60 mmHg at fraction of inspired oxygen of ≤ 0.4 with an extrinsic positive end-expiratory pressure (PEEP) <7 cmH_2_o; successful SBT of 60 min and treating physician approval for extubation; cardiovascular stability (i.e., absence of active myocardial ischemia, heart rate <130 beats per min, absence of vasopressor use, and dopamine or dobutamine <5 mcg/kg/min); body temperature between 36°C and 38°C; serum hemoglobin ≥8 g/dL; adequate coughing during suctioning and suction frequency of no more than every 2 h; and baseline cough observed by airway care score during suctioning (8). We excluded patients with missing baseline variables, those who failed SBT, those with tracheostomies, and those who withdrew all support or received comfort care support after extubation.

### Outcome Classification

Patients were grouped by ES or EF. ES was defined as the ability to maintain spontaneous unassisted respiration for ≥72 h after extubation. EF was defined as reintubation within 72 h after extubation.

### Data Collection

We collected data retrospectively by using Mayo Clinic ICU DataMart and Unified Data Platform, which are extensive data repositories that contain a near real time standardized replica of Mayo Clinic's EMR. These databases contain patient clinical information along with their laboratory test results, respiratory therapy notes, mechanical ventilation data, medications, vital sign flowsheets, and other clinical and pathological information from sources within the institution and have been previously validated (26). ICU Datamart stored validated ICU ventilator data at 15-min intervals and 2 min intervals in a surgical setting. The validation of ventilator data was done by the respiratory therapist and recorded in EMR.

Patient data collected included age, Acute Physiology and Chronic Health Evaluation score, mechanical ventilation duration, history of chronic comorbidities, hemoglobin and blood chemistries, arterial blood gas values within 1 h after onset of SBT, date and time of extubation, use of drugs (e.g., paralytics, systemic corticosteroids, etc.) during mechanical ventilation, negative inspiratory pressure measured prior to SBT, and GCS score assessed by the patient's nurse at the time of extubation. The airway care score (8) was recorded if available. Additionally, we collected data on respiratory variables including minute ventilation, respiratory rate, tidal volume, and at 1, 30, and 60 min during the SBT. We also collected RSBI recorded at 1 min (RSBI1), 30 min (RSBI30), and 60 min (RSBI60) during the SBT. We evaluated 2 pre-defined variables: (1) the RSBI30 to RSBI1 ratio as a percentage reflecting the change of RSBI from baseline to 30 min and (2) the RSBI60 to RSBI1 ratio as a percentage reflecting the change of RSBI from baseline to 60 min.

### Data Analysis

The main outcome of interest was EF. Categorical variables were reported as frequency and percentage and continuous variables as mean ± standard deviation (SD) and median (25th, 75th). We used the Wilcoxon rank sum test to compare continuous and ordinal variables between patients with and without EF, and χ^2^ or Fisher exact tests to compare categorical variable correlations. We used odds ratios (ORs) and 95% CIs to express a variable's strength for independently predicting EF in multivariate logistic regression models. Extreme outliers for variables ≥3 SD were verified manually in EMR. If the value was not available in the EMR, then we considered these erroneous values as missing in the final analysis. Probable predictor variables were chosen based on our clinical experience and information from other studies (11, 20, 27–36). Predictor variables that were significantly different between the success and failure groups (*P* ≤ 0.01) for which no more than 5% data were missing were included in multivariate analysis. Variable reduction was done based on the correlation between predictors and the threshold used was 0.6. Prediction scores were developed based on the multivariate logistic regression model coefficient estimate. The smallest coefficient was first identified and assigned a score of 1. Then the scores for the other variables were equal to their corresponding model coefficients divided by this smallest coefficient and finally, all scores were rounded to integers. We developed the RISC score by assigning an amount to each of the risk factors based on their model coefficients. Discrimination of the score as a continuous variable was reported as C-index. Area under the curve (AUC) was also reported for scores at different cutoff points. Calibration of the score was evaluated by calibration plot comparing the predicted and observed risk of EF within 72 h. All statistical tests were 2-sided, with an α-level of 0.05 for statistical significance. Analysis was done using SAS version 9.4 (SAS Institute Inc.).

## Results

The 6,161 patients included in our study were randomly allocated into a derivation set (*n* = 3,080) and validation set (*n* = 3,081). In the derivation set, patients had a mean (SD) age of 61.7 (16.6) years, and 1,820 (59%) were men. Within 72 h, 393 patients (12.8%) experienced EF. Similarly, in the validation set, the mean (SD) age was 62.4 (16.6) years and 1,778 (58%) were men. Within 72 h, 353 patients (11.5%) experienced EF (Table 1). Patient endotracheal secretions, mechanical ventilation, and ICU admission diagnosis data for both sets are displayed in Appendices A–C.

**Table 1:** Baseline demographic and hemodynamic instability and fluid status.

**Characteristic**	**Derivation (*n* = 3,080)**	**Validation (*n* = 3,081)**	**Total (*N* = 6,161)**	***P*-value**
Extubation failure	393	353	746	0.12
Age, y	
Mean (SD)	61.7 (16.6)	62.4 (16.6)	62.0 (16.6)	0.08
Median (IQR)	63.4 (51.9–74.0)	64.0 (52.2–75.1)	63.8 (52.1–74.5)	
Sex, No. (%)
Female	1,260 (40.9)	1,303 (42.3)	2,563 (41.6)	0.28
Male	1,820 (59.1)	1,778 (57.7)	3,598 (58.4)	
BMI (kg/m^2^), No. (%)	
Missing	17	21	38	0.84
<18.5	91 (3.0)	86 (2.8)	177 (2.9)	
18.5–24.9	771 (25.2)	755 (24.7)	1,526 (24.9)	
25.0–29.9	883 (28.8)	896 (29.3)	1,779 (29.1)	
30.0–34.9	648 (21.2)	624 (20.4)	1,272 (20.8)	
≥35.0	670 (21.9)	699 (22.8)	1,369 (22.4)	
GCS score prior to extubation	
Missing	0	0	0	0.64
Mean (SD)	9.6 (2.5)	9.6 (2.5)	9.6 (2.5)	
Median (IQR)	10 (8–11)	10 (8–11)	10 (8–11)	
Total RBC volume (mL) given within 24 h prior to extubation	
Missing	1	3	4	0.56
Mean (SD)	106.9 (366.6)	115.4 (402.9)	111.2 (385.2)	
Total platelet volume (mL) given within 24 h prior to extubation	
Missing	1	2	3	0.14
Mean (SD)	23.9 (117.4)	27.9 (133.4)	25.9 (125.7)	
Total cryoprecipitate volume (mL) given within 24 h prior to extubation	
Missing	0	2	2	0.80
Mean (SD)	3.9 (57.3)	2.4 (25.8)	3.1 (44.4)	
Total urine output (mL) within 24 h prior to extubation	
Missing	0	2	2	0.37
Mean (SD)	2,315.0 (1,713.7)	2,258.9 (1,634.8)	2,287.0 (1,674.8)	
Median (IQR)	2,043.5 (1,052.3–3,350.3)	2,025.0 (1,040.0–3,199.0)	2,036.0 (1,045.0–3,276.0)	
Fluid balance (mL) 24 h prior to extubation	
Missing	2	2	4	0.17
Mean (SD)	563.6 (2,924.4)	677.0 (2,970.5)	620.3 (2,947.9)	
Median (IQR)	274.2 (−1,061.9 to 1,601.1)	374.0 (−966.7 to 1,604.1)	318.1 (−1,013.6 to 1,601.1)	

*BMI, body mass index; GCS, Glasgow coma scale; IQR, interquartile range; RBC, red blood cell; SD, standard deviation*.

**Figure 2:**
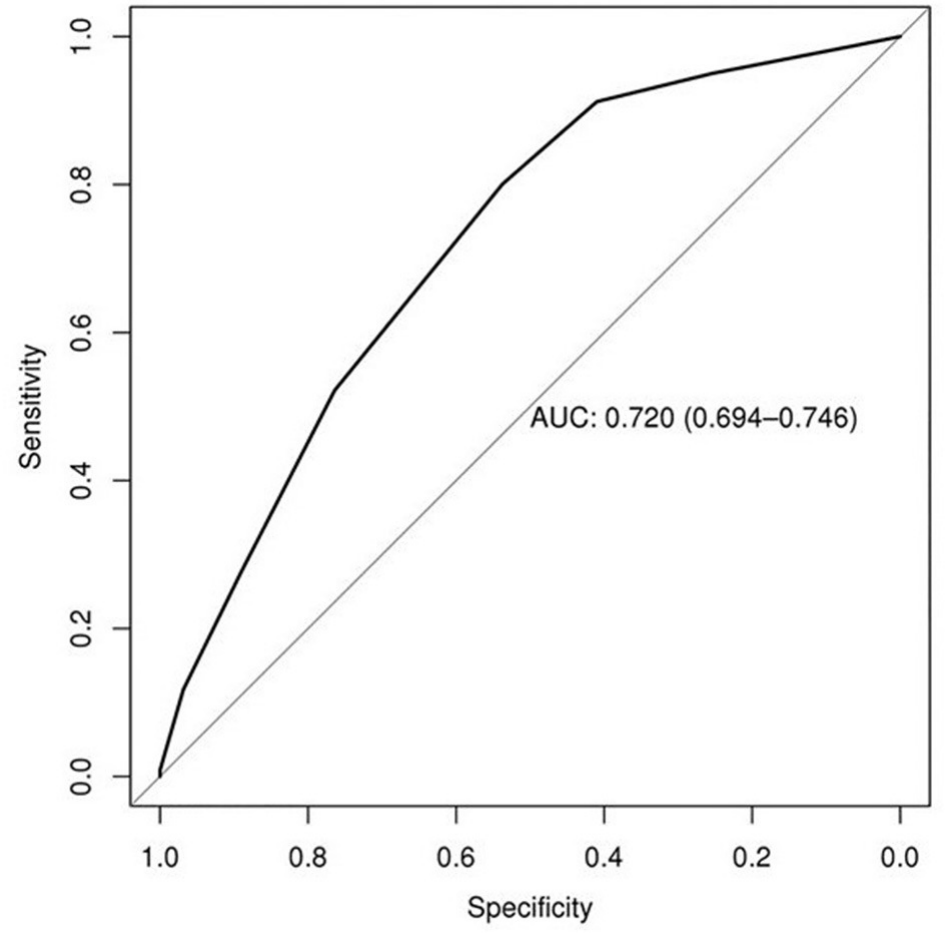
AUC receiver operating characteristic curve. AUC indicates area under the curve.

Predictors of EF in the derivation set included underweight status (body mass index, <18.5 kg/m^2^; OR, 1.91; 95% CI, 1.12–3.26; *P* = 0.02), GCS score of ≥10 (OR, 1.68; 95% CI, 1.31–2.16; *P* < 0.001), mean airway pressure (MAP) closest to 1 min after SBT start within 15 min <10 cmH_2_o (OR, 2.11; 95% CI, 1.68–2.66; *P* < 0.001), fluid balance of ≥1,500 mL 24 h prior to extubation (OR, 2.36; 95% CI, 1.87–2.96; *P* < 0.001), and mechanical ventilation ≥5 days (OR, 3.94; 95% CI, 3.04–5.11; *P* < 0.001) (Tables 2, 3). The derivation set had a C-index of 0.72 (95% CI, 0.70–0.75) (Figure 2).

**Table 2:** SBT and MV data for derivation and validation cohort.

**Characteristic**	**Derivation (*n* = 3,080)**	**Validation (*n* = 3,081)**	**Total (*N* = 6,161)**	***P*-value**
Total ventilation hours				
Missing	0	1	1	0.22
Mean (SD)	192.4 (194.4)	187.8 (189.7)	189.6 (191.9)	
Median (IQR)	131.4 (79.5–230.7)	127.6 (75.3–221.2)	129.0 (76.5–225.0)	
Respiratory rate (breaths/min) closest to 1 min after SBT start within 15 min				
Missing	16	16	32	0.49
Mean (SD)	20.6 (8.0)	20.4 (7.6)	20.5 (7.8)	
Median (IQR)	20.0 (15.0–25.0)	19.0 (15.0–24.4)	19.2 (15.0–25.0)	
Expired V_T_ (in ml) closest to 1 min after SBT start within 15 min				
Missing	123	130	253	0.34
Mean (SD)	517.9 (204.3)	512.6 (198.7)	515.3 (201.5)	
Median (IQR)	489.0 (389.0–620.0)	480.0 (386.0–601.0)	480.0 (388.0–610.0)	
Expired V_T_ (in ml/kg) closest to 1 min after SBT start within 15 min				
Missing	123	130	253	0.06
Mean (SD)	6.4 (2.7)	6.3 (2.7)	6.4 (2.7)	
Median (IQR)	6 (4.7–7.7)	5.9 (4.5–7.6)	5.9 (4.6–7.6)	
RSBI^a^ (breaths/min/L) closest to 1 min after SBT start within 15 min				
Missing	121	126	247	0.98
Mean (SD)	50.6 (44.3)	49.9 (41.3)	50.3 (42.9)	
Median (IQR)	40.0 (26.2–61.5)	40.8 (26.4–60.6)	40.4 (26.3–60.9)	
MAP (cmH_2_o) closest to 1 min after SBT start within 15 min				
Missing	31	45	76	0.75
Mean (SD)	10.7 (3.6)	10.8 (3.7)	10.8 (3.6)	
Median (IQR)	10.0 (7.8–13.0)	9.9 (7.8–13.0)	9.9 (7.8–13.0)	
PIP (cmH_2_o) closest to 1 min after SBT start within 15 min				
Missing	129	156	285	0.43
Mean (SD)	18.8 (6.4)	18.8 (6.2)	18.8 (6.3)	
Median (IQR)	18.0 (14.6–22.0)	18.0 (15.0–22.0)	18.0 (15.0–22.0)	
Plateau pressure (cmH_2_o) closest to 1 min after SBT start within 15 min				
Missing	1,028	1,074	2,102	0.92
Mean (SD)	18.3 (6.2)	18.2 (5.8)	18.3 (6.0)	
Median (IQR)	18.0 (14.0–21.0)	17.0 (15.0–21.0)	17 (14.0–21.0)	
PEEP (cmH_2_o) closest to 1 min after SBT start within 15 min				
Missing	0	0	0	0.53
Mean (SD)	7.5 (3.2)	7.5 (3.3)	7.5 (3.2)	
Median (IQR)	5 (5–10)	5 (5–10)	5 (5–10)	
PS (mmH_2_O) closest to 1 min after SBT start within 15 min				
Missing	6	9	15	0.96
Mean (SD)	8.9 (4.0)	8.8 (3.5)	8.9 (3.8)	
Median (IQR)	10 (5–10)	10 (5–10)	10 (5–10)	
History of paralytic use, No. (%)				
No	1,096 (35.6)	1,145 (37.2)	2,241 (36.4)	0.20
Yes	1,984 (64.4)	1,936 (62.8)	3,920 (63.6)	
History of sedative use, No. (%)				
No	202 (6.6)	232 (7.5)	434 (7.0)	0.15
Yes	2,878 (93.4)	2,849 (92.5)	5,727 (93.0)	

*IQR, interquartile range; MAP, mean airway pressure; MV, mechanical ventilation; PEEP, positive end–expiratory pressure; PIP, peak inspiratory pressure; PS, Pressure Support; RSBI, rapid shallow breathing index; SBT, spontaneous breathing trial; SD, Standard deviation; V_T_, tidal volume*.

a *RSBI, respiratory rate (f) in breaths/min/ V_T_ in L*.

**Table 3:** Logistic regression model predicting extubation failure.

		**Univariable**		**Multivariate**		
**Variable**	**Label**	**OR (95% CI)**	***P*-value**	**OR (95% CI)**	***P*-value**	**Score**
Underweight	Underweight	1.97 (1.18–3.27)	0.009	1.91 (1.12–3.26)	<0.02	1
GCS10	GCS score prior to CPAP mode ≥10	1.61 (1.27–2.05)	<0.001	1.68 (1.31–2.16)	<0.001	1
MAP10	MAP closest to 1 min after SBT start within 15 min <10	1.71 (1.38–2.13)	<0.001	2.11 (1.68–2.66)	<0.001	1
Fluid balance	Fluid balance 24 h prior to extubation ≥1,500	2.30 (1.85–2.86)	<0.001	2.36 (1.87–2.96)	<0.001	2
Ventday 5	Total ventilation days ≥5	3.54 (2.76–4.55)	<0.001	3.94 (3.04–5.11)	<0.001	3

*CPAP, continuous positive airway pressure; GCS, Glasgow coma scale; MAP, mean airway pressure; OR, odds ratio; SBT, spontaneous breathing trial*.

**Figure 3:**
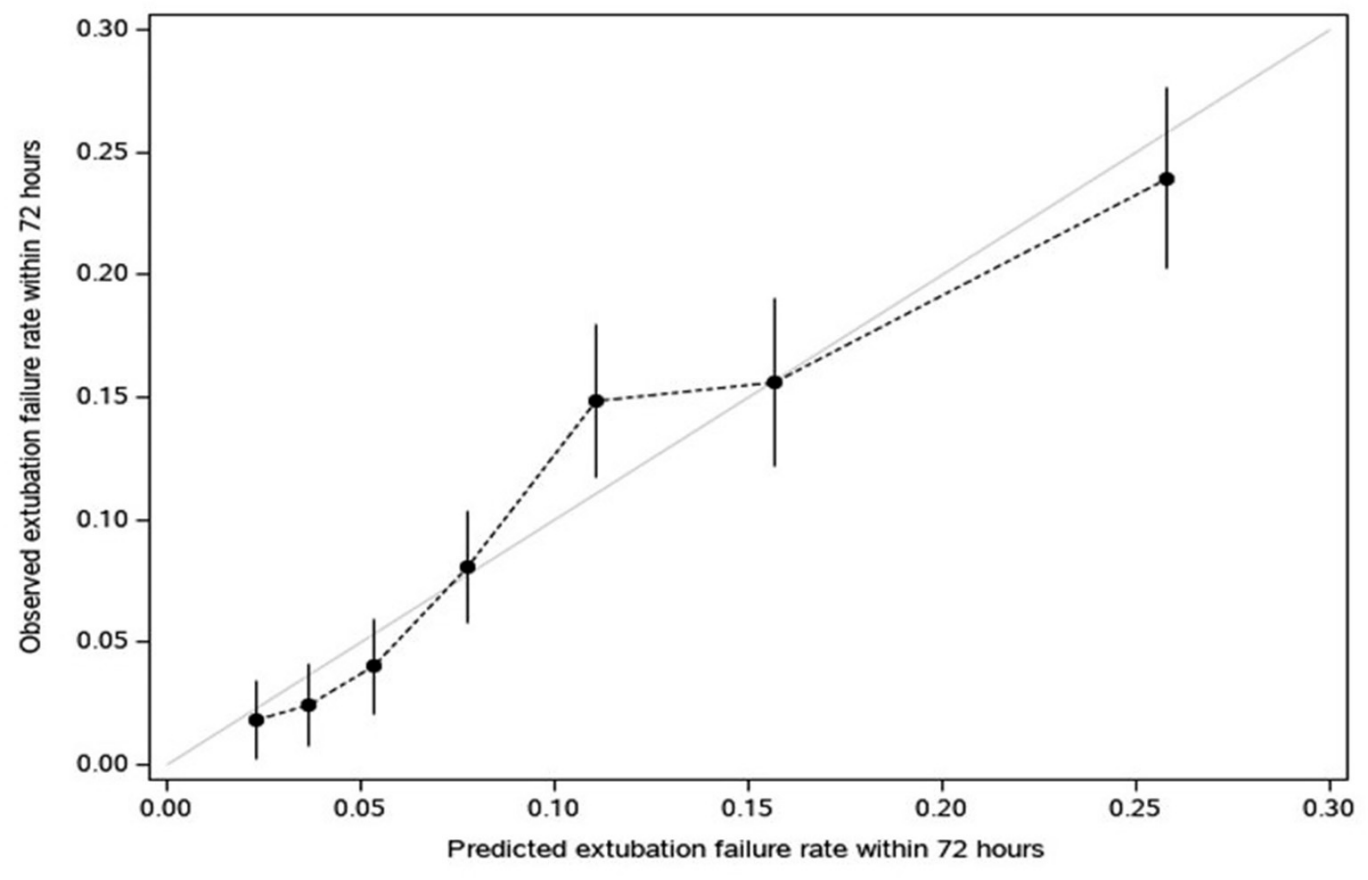
Calibration plot of observed vs. predicted extubation failure in the validation set (dots convey the apparent calibration from the original model, in which predictions for extubation failure within 72 h are grouped into deciles (10 groups ranging from low to high likelihood) and each related to observed rates (vertical bars reflect 95% CI for the rate); For a reference of perfect calibration, the Y = X line is displayed).

**Figure 4:**
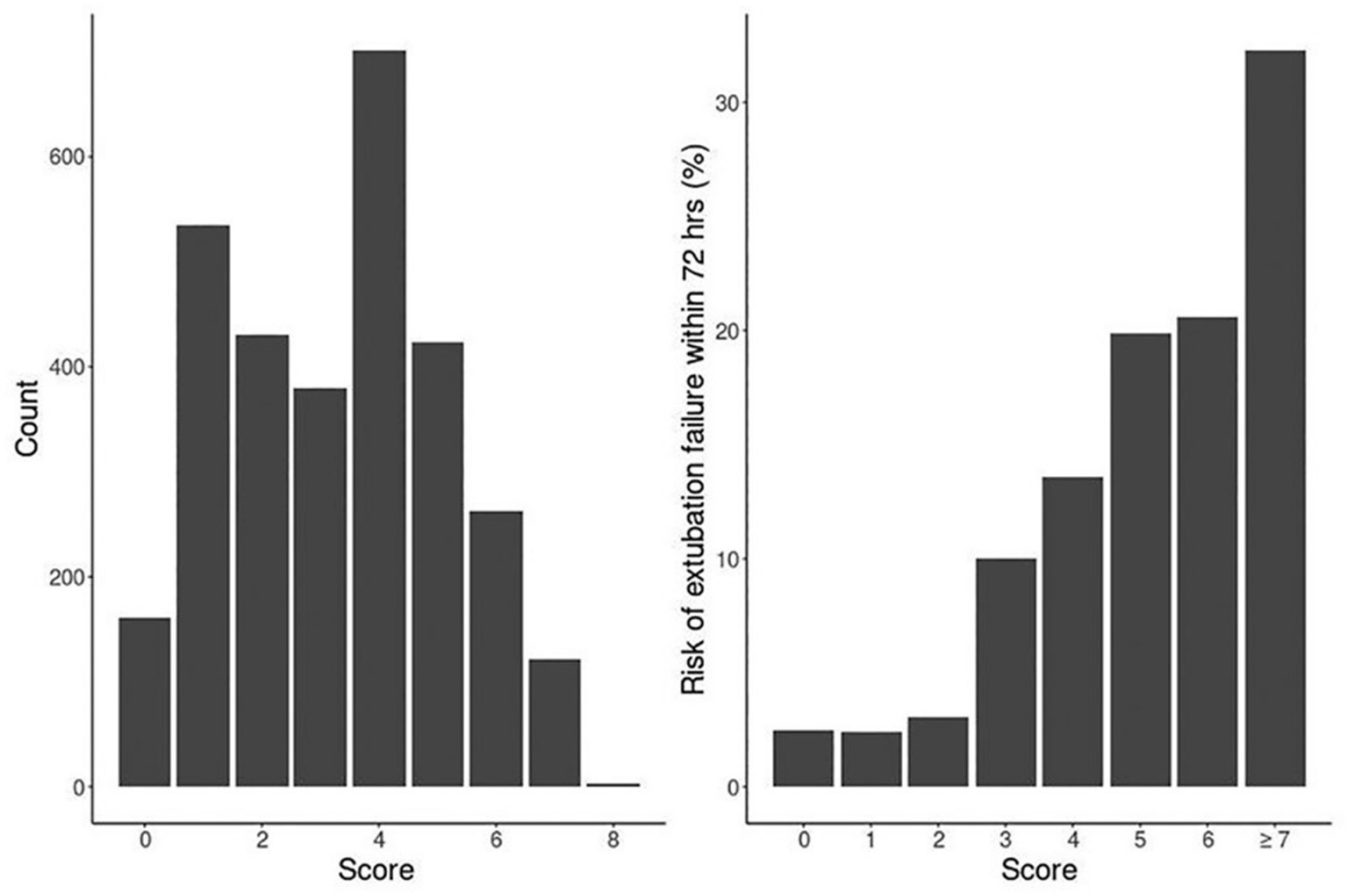
Distribution of RISC score and the corresponding 72-h extubation failure rate (%).

Our logistic model in validation set demonstrated that as RISC increased by 1, the odds of having EF became 1.6-fold higher (OR, 1.58; 95% CI, 1.47–1.69; *P* ≤ 0.001) (Table 4). Receiving operating curve analysis revealed the best cutoff for RISC was 4, which demonstrated a sensitivity of 0.80 and specificity of 0.54 with AUC of 0.67 (95% CI, 0.65–0.69) (Appendix C). Calibration plot of observed vs. predicted EF in the validation set is displayed in Figure 3. Using the above model, the validation set had a C-index of 0.72 (95% CI, 0.69–0.75). The RISC score ranged from 0 to 8 with a median of 4 (Figure 4).

**Table 4:** Validation set: extubation within 72 h predicted by score.

**Variable**	**OR (95% CI)**	***P*-value**	**C-index**
RISC score	1.58 (1.47–1.69)	<0.001	0.72

*OR, odds ratio; RISC, Reintubation Summation Calculation*.

## Discussion

We successfully developed a multivariable RISC score to predict extubation failure after a successful SBT with readily available bedside predictors. The RISC score predicts extubation failure with the best cut-off at ≥4 demonstrating a sensitivity of 0.80 and a specificity of 0.54 with AUC of 0.67. This is a modest value to determine extubation failure. RISC score provides several multivariate risk factors that can be externally validated in future deep learning and machine learning predictive models. We acknowledge this model is limited and some would argue that a clinically useful tool should have a higher AUC; however, external testing with larger data sets may be helpful in reproducing these results.

Neurologic impairment was found to be a possible risk factor for EF in our study and was validated previously in several studies (16, 19, 37–39). Mokhlesi et al. (37) verified that a moderate GCS score (9–12) can clinically predict reintubation, comparable to our results. We feel this is an important finding for clinicians caring for those patients with an “intermediate” GCS in their decision-making for extubation. GCS is inherently limited in finding lower cutoffs since a “V1-NT” is the subcomponent reported in intubated patients. Moreover, its components only grade Eyes (E1-4) and Motor (M1-6) which if maximal total 10 points (E4+M6), similar to our observed lowest cutoff. Some suggest using the **F**ull **O**utline of **U**n**R**esponsiveness (FOUR) score (40) might provide a more granular range (0–16) by including brainstem cranial nerve findings. We reviewed our data for FOUR score (40), but only had 228 patients, which is statistically underpowered to detect lower limits of this coma scale for reintubation risk. Only a future study using FOUR score with sufficient sample size may be able to detect this as a true reintubation risk, or in a future randomized trial in brain-injured patients similar to the one performed by WDF, which previously showed no difference in reintubation rates in GCS 10 or less patients with intact brainstem protective reflexes of cough/gag (9). We find documentation of protective airway or cranial nerve reflexes lacking in most and/or all coma scales, even in the FOUR score which focuses on pupillary reflexes for the brainstem. Furthermore, the FOUR score did not include gag/cough reflexes in the randomized trial by Manno et al. (9).

Another covariate of the RISC score is a MAP closest to 1 min after SBT start within 15 min <10 cmH_2_o. MAP is dependent on peak inspiratory pressure, PEEP, and respiratory cycle time. Our finding of low MAP leading to extubation failure may be explained by a dyspneic patient with vigorous efforts. Mean airway pressure is a pressure monitoring metric used by mechanical ventilators that are closely connected to mean alveolar pressure and depict pressures on the lung parenchyma during ventilation (41). It's also connected to the oxygenation index (42). Peak inspiratory pressure, PEEP, and the inspiratory-to-expiratory time ratio with dynamic and real-time features are used to calculate MAP, which measures mechanical power impacted by the ventilator mode (43). A high MAP suggests that the patient's mechanical energy power is greater (43). Furthermore, MAP is a critical pressure parameter that influences a patient's hemodynamics. It has been established that higher MAP causes a reduction in cardiac output in infants during both normal and high-frequency mechanical ventilation (44, 45). In direct proportion to their effects on MAP, tidal forces and PEEP raise pulmonary vascular resistance (PVR) (46). As a result, MAP is becoming more linked to the prognosis of patients on mechanical ventilation. A patient that is not able to mount a high MAP may demonstrate insufficient respiratory mechanics from possibly, diaphragmatic weakness. Patients who are able to mount a high MAP may have more strength in respiratory muscles, and thus mechanics, thereby resulting in tolerance of extubation. In summary, we argue that MAP has several strengths over other conventional mechanical ventilator parameters especially pressure indicators, and it has the ability to become a major bridge factor coupling respiratory mechanics and hemodynamics. However, we acknowledge there are factors potentially not assessed in this study during the transition from positive to negative pressure physiology, stressors on cardiac physiology, and potentially Co_2_ accumulation that could theoretically occur before extubation.

Thille et al. (33) identified prolonged mechanical ventilation (duration <1 week) prior to weaning as a strong predictor for EF, similar to our results. We observed that underweight status was also associated with EF and an important factor since it underlies neuromuscular reserve. Malnutrition is reported in as much as 40% of critically ill patients, but data linking nutritional status to ventilator weaning issues are limited (47, 48). In the acute respiratory distress syndrome (ARDS) network trial (43); 4.7% of patients were identified as underweight. Underweight patients, defined as those with a body mass index <18.5 kg/m^2^, may experience depressed ventilatory drive (49), limited muscle mass (50), and weaning difficulty (51). Obesity may be associated with a better prognosis (“obesity paradox”) for some disease states, patients with ARDS (52) and those in the ICU in general (53, 54). Another important covariate in EF was positive fluid balance, as reported by Frutos-Vivar et al. (27). This is an important finding for clinicians to review prior to extubation attempts since diuretics could be given in future RISC-driven randomized trials on EF. D'Orio et al. (55) reported that a positive cumulative fluid balance may cause increased capillary leak and extravascular lung water and decreased lung compliance, leading to respiratory failure, both during SBT and in the immediate post-extubation period. Hence why restrictive fluid strategies are employed in ARDS patients (56). In an earlier study, positive cumulative fluid balance from hospital admission to weaning was correlated with EF (57). Therefore, it is highly plausible that positive fluid balance influences the respiratory outcome of patients.

To our knowledge, there are no comprehensive data sets analyzing all the variables we proposed in predicting ES, particularly with neurologic components. Most studies focus on RSBI as a predictor in the post-operative setting (27, 37, 58). RSBI is challenging however in patients in pain with tachypnea and some neurologic patients with abnormal brain-disordered breathing states. The closest study we found in the literature similar to ours was a neonatal extubation modeling study by Mueller et al. (54), who used artificial neural-networks, receiver operating characteristics, and regression modeling to predict ES. The authors looked at inspiratory to expiratory ratio, inspiratory time, MAP, tidal volume, and Sao_2_. The authors found an AUC of 0.87, which is a fairly strong prediction for ES (59). Other studies have studied ES but these have been largely focused on operating room predictors and not global predictors (60). Rodriguez Blanco et al. (60) studied 78 surgery patients who had adequate ejection fraction and other standard clinical factors. This study did not adequately characterize the respiratory variables proposed and was of relatively small sample size. Similarly, data from a cohort of mechanically ventilated elderly patients were prospectively analyzed and used to develop a predictive model using a classification and regressive tree (CART) algorithm, also known as a decision tree to predict extubation outcome in patients following a successful SBT (29, 30). This CART model showed a good discrimination with an AUC of 0.94. However, calibration was moderate with a substantial mismatch between predicted and actual probabilities in the updated CART model (47).

Our study has several limitations. First, the retrospective design limits extrapolation to prospective and individualized patient care contexts. Retrospective studies can introduce selection and information biases. We were also not able to collect data on all clinical weaning predictors such as diaphragm movement, endotracheal secretions, and hypercapnic ventilatory response, Airway occlusion pressure in each patient. Second, our study lacked granular data on supplemental oxygen strategies post-extubation as recent literature supports high flow nasal cannula combined with non-invasive in preventing re-intubation (61, 62). Third, the study was completed at a single center with retrospective data collection, and thus does not allow inferences on causality derived from prospective data. Fourth, we were not able to collect echocardiographic measurements to be able to correlate positive fluid balance with ventricular dysfunction. Fifth, in our model, there may be some lack of variability using a retrospective cohort from the same center, and thus validation and accuracy may be overestimated. Sixth, some of the ventilator data captured in this study such as the reporting of a plateau pressure measurement during pressure support in a spontaneously breathing patient were from Puritan Bennett 840 ventilators (Medtronic). These ventilators record into the EMR what it believes are plateau pressures in any mode, even when an adequate inspiratory pause is absent. We have documented instances where vitals are being recorded at greater than the 15-min standard of the time for procedures or more while ventilator settings remained as they were. Lastly, our multivariate covariates within our RISC score model are generally known single risk factors of EF in the existing literature (27, 28, 33–35, 63). Therefore, our study should be considered with caution as exploratory only and requires prospective and external validation of the multivariate model before implementing into routine clinical decision making. This is especially true given data is from a single site that may reflect a unique culture not practiced by others.

Despite our limitations, this study has several strengths. A major strength is that it was done using a robust and clean dataset derived from a previously validated database (64). The study also included a large cohort of patients from a large, tertiary academic hospital. To date, there are no comprehensive data sets analyzing all the variables in predicting ES, especially with neurological components; therefore, we feel this study could represent the first adult human modeling study, and by the addition of more variables, an even more potentially accurate and precise model for predicting ES in the future. However, we still recommend external validation before generalizing and implementing these results as predictive models.

## Conclusions

We developed the RISC score using several practical clinical parameters tested within a derivation and validation set. This model provides risk-stratification for extubation and subsequent EF within 72 h in mechanically ventilated patients in the ICU. Overall, we identified 5 predictors of EF readily available at the bedside and in many EMRs: underweight status (body mass index <18.5 kg/m^2^), GCS score ≥10, MAP closest to 1 min after SBT start within 15 min <10 cmH_2_o, fluid balance of ≥1,500 mL 24 h prior to extubation, and mechanical ventilation ≥5 days. External validation in a larger, multicenter study is required before clinical implementation.

## Data Availability Statement

The original contributions presented in the study are included in the article/Supplementary Material, further inquiries can be directed to the corresponding author/s.

## Ethics Statement

The studies involving human participants were reviewed and approved by Mayo Clinic Institutional Review Board. Written informed consent for participation was not required for this study in accordance with the national legislation and the institutional requirements.

## Disclosure

Presented at the Neurocritical Care Society 15th Annual Meeting, Waikoloa Village, Hawaii, October 13-17, 2017 and portions of this manuscript have been published in abstract form and available online at https://link.springer.com/journal/12028/volumes-and-issues/27-2/supplement and citation “Bansal V, Li Z, Marquez A, Kashyap R, Smelick CP, Diaz-Gomez JL, et al. **R**e-**I**ntubation **S**cale **C**alculation (**RISC**): predicting extubation failure in critically ill patients. *Neurocritical Care*. (2017) 27(Suppl. 2):S122.”

## Author Contributions

VB, NS, RK, and WF contributed to the conception and design of the study. VB and AM organized the database. VB and ZL performed the statistical analysis. All authors contributed to the article and approved the submitted version.

## Funding

This work was supported by the Department of Neurology at Mayo Clinic, Jacksonville, Florida with no direct funding.

## Conflict of Interest

The authors declare that the research was conducted in the absence of any commercial or financial relationships that could be construed as a potential conflict of interest.

## Publisher's Note

All claims expressed in this article are solely those of the authors and do not necessarily represent those of their affiliated organizations, or those of the publisher, the editors and the reviewers. Any product that may be evaluated in this article, or claim that may be made by its manufacturer, is not guaranteed or endorsed by the publisher.

## Abbreviations

ARDS: acute respiratory distress syndrome
AUC: area under the curve
EF: extubation failure
ES: extubation success
FOUR: Full Outline of UnResponsiveness
GCS: Glasgow coma scale
ICU: intensive care unit
MAP: mean airway pressure
OR: odds ratio
RISC: Reintubation Scale Calculation
RSBI: rapid shallow breathing index
SBT: spontaneous breathing trial.
