# Supplementary material for: Reintubation Summation Calculation: A Predictive Score for Extubation Failure in Critically Ill Patients
Source: Front Med (Lausanne). 2022 Feb 17;8:789440. doi: 10.3389/fmed.2021.789440 (PMC8891541; doi:10.3389/fmed.2021.789440)
Supplement: Supplementary file 1 [file Table_1.docx]

**Appendix A. Endotracheal Secretions (Airway Care Score)**

|  | **Derivation (n=3080)** | **Validation (n=3081)** | **Total (N=6161)** | ***P* value** |
| --- | --- | --- | --- | --- |
| **Extubation Failure** | **n=393** | **n=353** | **n=746** |  |
| **ACS closest to one minute after SBT start within 15 minutes** | | | | |
| N | 1776 | 1752 | 3528 | 0.51 |
| Missing | 1304 | 1329 | 2633 |  |
| Mean (SD) | 3.6 (2.0) | 3.6 (2.0) | 3.6 (2.0) |  |
| **Score of Cough amount charted closest to one minute after SBT start within 15 minutes** | | | | |
| N | 222 | 218 | 440 | 0.76 |
| Missing | 2858 | 2863 | 5721 |  |
| Mean (SD) | 1.2 (1.3) | 1.2 (1.3) | 1.2 (1.3) |  |
| **Score of Sputum amount charted closest to one minute after SBT start within 15 minutes** | | | | |
| N | 453 | 462 | 915 | 0.25 |
| Missing | 2627 | 2619 | 5246 |  |
| Mean (SD) | 1.6 (0.8) | 1.7 (0.7) | 1.6 (0.8) |  |
| **Score of Sputum color charted closest to one minute after SBT start within 15 minutes** | | | | |
| N | 440 | 451 | 891 | 0.15 |
| Missing | 2640 | 2630 | 5270 |  |
| Mean (SD) | 1.2 (0.8) | 1.1 (0.7) | 1.1 (0.7) |  |
| **Score of Sputum Viscosity charted closest to one minute after SBT start within 15 minutes** | | | | |
| N | 432 | 441 | 873 | 0.39 |
| Missing | 2648 | 2640 | 5288 |  |
| Mean (SD) | 1.5 (0.9) | 1.4 (0.9) | 1.5 (0.9) |  |

Abbreviations: ACS: Airway care score, SBT: spontaneous breathing trial, SD: Standard deviation
