# Supplementary material for: Reintubation Summation Calculation: A Predictive Score for Extubation Failure in Critically Ill Patients
Source: Front Med (Lausanne). 2022 Feb 17;8:789440. doi: 10.3389/fmed.2021.789440 (PMC8891541; doi:10.3389/fmed.2021.789440)
Supplement: Supplementary file 2 [file Table_2.docx]

**Appendix B. Patient Characteristics (Comorbidities, Reason for ICU admission, ICU mortality and Hospital Mortality)**

|  | **Derivation (n=3080)** | **Validation (n=3081)** | **Total (N=6161)** | **P value** |
| --- | --- | --- | --- | --- |
| **Comorbidities** | | | | |
| **Hypertension** | 1263 (41.0%) | 1278 (41.5%) | 2541 (41.2%) | 0.72 |
| **Coronary Artery Disease** | 590 (19.2%) | 582 (18.9%) | 1172 (19.0%) | 0.80 |
| **Chronic Kidney Disease** | 446 (14.5%) | 458 (14.9%) | 904 (14.7%) | 0.69 |
| **Chronic Liver Disease** | 64 (2.1%) | 51 (1.7%) | 115 (1.9%) | 0.22 |
| **Diabetes** | 587 (19.1%) | 548 (17.8%) | 1135 (18.4%) | 0.20 |
| **COPD** | 329 (10.7%) | 376 (12.2%) | 705 (11.4%) | 0.07 |
| **Cancer** | 73 (2.4%) | 74 (2.4%) | 147 (2.4%) | 1 |
| **CHF** | 638 (20.7%) | 627 (20.4%) | 1265 (20.5%) | 0.72 |
| **Valvular Heart Disease** | 617 (20.0%) | 608 (19.7%) | 1225 (19.9%) | 0.77 |
| **Reason for ICU admission** | | | | |
| **ARDS** | 83 (2.7%) | 79 (2.6%) | 162 (2.6%) | 0.75 |
| **Stroke** | 163 (5.3%) | 204 (6.6%) | 367 (6.0%) | 0.03 |
| **Cerebral disease other** | 69 (2.2%) | 80 (2.6%) | 149 (2.4%) | 0.36 |
| **Septic Shock** | 192 (6.2%) | 186 (6.0%) | 378 (6.1%) | 0.75 |
| **Cardiac Arrest** | 257 (8.3%) | 244 (7.9%) | 501 (8.1%) | 0.54 |
| **COPD Exacerbation** | 193 (6.3%) | 206 (6.7%) | 399 (6.5%) | 0.50 |
| **Pneumonia** | 842 (27.3%) | 787 (25.5%) | 1629 (26.4%) | 0.11 |
| **Dysrhythmia** | 926 (30.1%) | 935 (30.3%) | 1861 (30.2%) | 0.81 |
| **Acute CHF Exacerbation** | 143 (4.6%) | 161 (5.2%) | 304 (4.9%) | 0.29 |
| **Pulmonary Embolism** | 178 (5.8%) | 173 (5.6%) | 351 (5.7%) | 0.78 |
| **Acute Renal Failure** | 720 (23.4%) | 698 (22.7%) | 1418 (23.0%) | 0.50 |
| **Gastrointestinal Hemorrhage** | 155 (5.0%) | 154 (5.0%) | 309 (5.0%) | 0.95 |
| **Cardiogenic Shock** | 626 (20.3%) | 624 (20.3%) | 1250 (20.3%) | 0.94 |
| **Neurogenic Shock** | 284 (9.2%) | 282 (9.2%) | 566 (9.2%) | 0.93 |
| **Mortality Outcomes** | | | | |
| **ICU Mortality** | 288 (9.4%) | 260 (8.4%) | 548 (8.9%) | 0.21 |
| **Hospital Mortality** | 539 (17.5%) | 526 (17.1%) | 1065 (17.3%) | 0.66 |

Abbreviations: ARDS, acute respiratory distress syndrome; COPD, chronic obstructive pulmonary disease; ICU, intensive care unit, CHF, Congestive Heart Failure
