# Supplementary material for: Reintubation Summation Calculation: A Predictive Score for Extubation Failure in Critically Ill Patients
Source: Front Med (Lausanne). 2022 Feb 17;8:789440. doi: 10.3389/fmed.2021.789440 (PMC8891541; doi:10.3389/fmed.2021.789440)
Supplement: Supplementary file 3 [file Table_3.docx]

**Appendix C. Validation Set: ROC Analysis at Different Score Cutoff Points***

| **Performance** | **Score ≥1** | **Score ≥2** | **Score ≥3** | **Score ≥4** | **Score ≥5** | **Score ≥6** | **Score ≥7** |
| --- | --- | --- | --- | --- | --- | --- | --- |
| Sensitivity | 98.83 | 95.01 | 91.20 | 80.06 | 52.20 | 27.57 | 11.73 |
| Specificity | 5.87 | 25.39 | 40.99 | 53.74 | 76.40 | 89.08 | 96.86 |
| PPV | 11.81 | 13.97 | 16.46 | 18.08 | 22.00 | 24.35 | 32.26 |
| NPV | 97.52 | 97.56 | 97.33 | 95.48 | 92.61 | 90.60 | 89.59 |
| Accuracy | 16.38 | 33.27 | 46.67 | 56.72 | 73.67 | 82.12 | 87.23 |
| AUC | 0.52 | 0.60 | 0.66 | 0.67 | 0.64 | 0.58 | 0.54 |

Note: ^*^Data reports as percentages.

Abbreviation: AUC, area under the ROC Curve; NPV, negative predictive value; PPV, positive predictive value
