# Supplementary material for: Reintubation Summation Calculation: A Predictive Score for Extubation Failure in Critically Ill Patients
Source: Front Med (Lausanne). 2022 Feb 17;8:789440. doi: 10.3389/fmed.2021.789440 (PMC8891541; doi:10.3389/fmed.2021.789440)
Supplement: Supplementary file 4 [file Table_4.docx]

**Appendix D. TRIPOD Checklist: Prediction Model Development and Validation**

| **Section/Topic** | **Checklist Item** | **Page** |
| --- | --- | --- |
| **Title and abstract** | | |
| Title | Identify the study as developing and/or validating a multivariable prediction model, the target population, and the outcome to be predicted. | 1 |
| Abstract | Provide a summary of objectives, study design, setting, participants, sample size, predictors, outcome, statistical analysis, results, and conclusions. | 1 |
| **Introduction** | | |
| Background and objectives | Explain the medical context (including whether diagnostic or prognostic) and rationale for developing or validating the multivariable prediction model, including references to existing models. | 2 |
|  | Specify the objectives, including whether the study describes the development or validation of the model or both. |  |
| **Methods** | | |
| Source of data | Describe the study design or source of data (e.g., randomized trial, cohort, or registry data), separately for the development and validation data sets, if applicable. | 2-3 |
|  | Specify the key study dates, including start of accrual; end of accrual; and, if applicable, end of follow-up. |  |
| Participants | Specify key elements of the study setting (e.g., primary care, secondary care, general population) including number and location of centres. |  |
|  | Describe eligibility criteria for participants. |  |
|  | Give details of treatments received, if relevant. |  |
| Outcome | Clearly define the outcome that is predicted by the prediction model, including how and when assessed. |  |
|  | Report any actions to blind assessment of the outcome to be predicted. |  |
| Predictors | Clearly define all predictors used in developing or validating the multivariable prediction model, including how and when they were measured. |  |
|  | Report any actions to blind assessment of predictors for the outcome and other predictors. |  |
| Sample size | Explain how the study size was arrived at. |  |
| Missing data | Describe how missing data were handled (e.g., complete-case analysis, single imputation, multiple imputation) with details of any imputation method. |  |
| Statistical analysis methods | Describe how predictors were handled in the analyses. |  |
|  | Specify type of model, all model-building procedures (including any predictor selection), and method for internal validation. |  |
|  | For validation, describe how the predictions were calculated. |  |
|  | Specify all measures used to assess model performance and, if relevant, to compare multiple models. |  |
|  | Describe any model updating (e.g., recalibration) arising from the validation, if done. |  |
| Risk groups | Provide details on how risk groups were created, if done. |  |
| Development vs. validation | For validation, identify any differences from the development data in setting, eligibility criteria, outcome, and predictors. |  |
| **Results** | | |
| Participants | Describe the flow of participants through the study, including the number of participants with and without the outcome and, if applicable, a summary of the follow-up time. A diagram may be helpful. | Page 3-4, Table 1,  Table 2,  Table 3,  Table 4,  Figure 1,  Figure 2,  Figure 3,  Figure 4 |
|  | Describe the characteristics of the participants (basic demographics, clinical features, available predictors), including the number of participants with missing data for predictors and outcome. |  |
|  | For validation, show a comparison with the development data of the distribution of important variables (demographics, predictors and outcome). |  |
| Model development | Specify the number of participants and outcome events in each analysis. |  |
|  | If done, report the unadjusted association between each candidate predictor and outcome. |  |
| Model specification | Present the full prediction model to allow predictions for individuals (i.e., all regression coefficients, and model intercept or baseline survival at a given time point). |  |
|  | Explain how to the use the prediction model. |  |
| Model performance | Report performance measures (with CIs) for the prediction model. |  |
| Model-updating | If done, report the results from any model updating (i.e., model specification, model performance). |  |
| **Discussion** | | |
| Interpretation | For validation, discuss the results with reference to performance in the development data, and any other validation data. | 4-8 |
|  | Give an overall interpretation of the results, considering objectives, limitations, results from similar studies, and other relevant evidence. |  |
| Implications | Discuss the potential clinical use of the model and implications for future research. |  |
| Limitations | Discuss any limitations of the study (such as non-representative sample, few events per predictor, missing data). |  |
| **Other information** | | |
| Supplementary information | Provide information about the availability of supplementary resources, such as study protocol, Web calculator, and data sets. | Appendix A to Appendix D |
| Funding | Give the source of funding and the role of the funders for the present study. | 9 |
